# Up-regulated and interrelated expressions of GINS subunits predict poor prognosis in hepatocellular carcinoma

**DOI:** 10.1042/BSR20181178

**Published:** 2018-12-07

**Authors:** Yi-Fan Lian, Shan-Shan Li, Yan-Lin Huang, Huan Wei, Dong-Mei Chen, Jia-Liang Wang, Yue-Hua Huang

**Affiliations:** 1 Guangdong Provincial Key Laboratory of Liver Disease Research, The Third Affiliated Hospital of Sun Yat-sen University, Guangzhou, China; 2 Department of Medical Oncology, The Sixth Affiliated Hospital of Sun Yat-sen University, Guangzhou, China; 3 Department of Infectious Diseases, The Third Affiliated Hospital of Sun Yat-sen University, Guangzhou, China

**Keywords:** bioinformatics, GINS, hepatocellular carcinoma, prognosis, survival

## Abstract

The GINS complex is one of the core components of the eukaryotic replicative helicase CMG (Cdc45–MCM helicase–GINS) complex that serves as the replicative helicase unwinding duplex DNA ahead of moving replication fork during chromosome duplication. Many studies have highlighted the important functions amongst GINS subunits in various cancers. Nevertheless, the functions and prognostic roles of distinct GINS subunits in hepatocellular carcinoma (HCC) were largely unexplored. In the present study, we reported the prognostic values of GINS subunits in HCC patients through analysis of several databases, including Oncomine, (TCGA), and Kaplan–Meier Plotter (KMPlotter). We found that mRNA expressions of all GINS subunits were significantly up-regulated in HCC tumor than in non-tumor liver tissues. Survival analysis revealed that elevated expression of individual GINS subunit predicts a poor overall survival (OS) in all HCC patients. When sorting the patients by gender, the correlation between elevated expression of individual GINS subunit and poor OS remains significant in male patient subgroup, but not in female patient subgroup. Additionally, we found that co-overexpression of all GINS subunits was significantly associated with a higher hazard ratio, suggesting the GINS complex may co-operate to promote HCC progression. Indeed, their expressions were highly correlated with each other in the same cohort and TRANSFAC analysis revealed that four transcription factors including C/EBPα, Oct-1, Sp1, and USF may serve as common transcription factors binding to the promoters of all four GINS subunits. Therefore, we propose that individual GINS subunit or GINS complex as a whole could be potential prognostic biomarkers for HCC.

## Introduction

High-fidelity genomic DNA replication is important to all forms of cellular life and requires the complex interplay of various protein factors in a spatially and temporally regulatory manner [1]. DNA helicases unwind or rearrange duplex DNA during replication, recombination, and repair, thus playing an important role in preservation of genome stability. Abnormal helicase function leads to failure of faithful replication of DNA and thereafter the loss of genome integrity, which may be the cause of human cancers or diseases related to carcinogenesis [2]. Therefore, DNA helicases may have close relationship to and be considered as potential targets for human cancers.

The GINS complex (named after the four related subunits of the complex Sld5, Psf1, Psf2, and Psf3 from the Japanese go-ichi-ni-san meaning 5-1-2-3, corresponding to GINS4, GINS1, GINS2, and GINS3 in human genome, respectively) was a core component of the eukaryotic replicative helicase CMG (Cdc45–MCM helicase–GINS) complex that serves as the replicative helicase unwinding duplex DNA ahead of moving replication fork during chromosome duplication [3–5]. Previous report suggested that the four subunits of human GINS form a 100-kDa heterotetrameric complex with 1:1:1:1 stoichiometry and show no observable enzymatic activity [6]. During the initiation and elongation stages of replication, the GINS complex binds to and enhances the enzymatic function of MCM helicase, and also by forming the CMG complex provides a basis for recruiting other essential factors to generate a larger protein apparatus called replisome progression complex [7].

Overexpression of each GINS subunit has been reported in different human cancers. Higher expression level of GINS1 was observed in breast and lung cancer cells, and was correlated to enhanced ability in tumor proliferation and poor patient survival [8,9]. GINS2 expression was increased in intrahepatic cholangiocarcinoma and early-stage cervical cancer [10,11]. GINS3 expression was increased in colon carcinoma and lung cancer, and served as an unfavorable prognostic biomarker for cancer patients [12,13]. GINS4 expression was increased in bladder cancer and many cancer cell lines [14,15]. Additionally, inhibition of the GINS expression by RNA interfering reduces proliferation in HeLa cells [16] or impairs migration and invasion in breast cancer cells [17]. Therefore, the GINS subunits can act not only as prognostic biomarkers but also as druggable targets for cancer development and progression.

Hepatocellular carcinoma (HCC) is the most common primary liver malignancy, leading to approximately 700000 deaths per year [18]. Further studies of HCC oncogenes would help to identify novel molecular markers of HCC progression and develop new diagnostic and therapeutic strategies. Overexpressing DNA helicases including RUVBL2 [19], RecQL1 [20], and RecQL4 [21] had been shown to enhance cancer cell viability and were associated with poor patient survival in HCC, arguing an important role of DNA helicase in liver carcinogenesis. With respect to GINS complex, only one report has stated the correlation between GINS1 expression and HCC [22]. However, the prognostic roles of other GINS subunits in HCC remain largely unknown. In the current study, we extended the research field based on different databases in order to identify the prognostic values of individual GINS subunits or as a whole in HCC.

## Methods

### Oncomine analysis

The mRNA levels of distinct GINS subunits in diverse cancer types were determined through analysis in ONCOMINE database (www.oncomine.org), which is a publicly accessible online database with cancer microarray information to facilitate discovery from genome-wide expression analyses. In the present study, Student’s *t*test generated *P*-value for transcriptional expression difference between datasets derived from cancer specimens and normal controls. The fold change was defined as >1 and *P*-value was set up at 1E-04.

### The Cancer Genome Atlas dataset

We used the FireBrowse (http://firebrowse.org/) to obtain The Cancer Genome Atlas (TCGA) liver cancer data, which were used to evaluate the mRNA expressions of GINS subunits in different histological grades and the correlations between each other in human HCC. Non-parametric Mann–Whitney test was used to analyze the differences of all GINS expressions between tumor and normal liver tissue or HCC cohorts of different histological grades. The associations between expression levels of GINS subunits were analyzed by Spearman’s rank test. *P*-value <0.05 was considered statistically significant.

### The Kaplan–Meier Plotter survival analysis

The prognostic value of individual GINS subunit was analyzed using an open online database Kaplan–Meier Plotter (KMPlotter) (http://kmplot.com/analysis/) that established using gene expression data and survival information of liver cancer and other four types of cancer including breast cancer, ovarian cancer, lung cancer, and gastric cancer. The desired probe ID was determined according to the file of probe sets provided by KMPlotter. Briefly, each GINS subunit was entered into database with or without specific restrictions like Gender or Grade of HCC. The cancer patients were divided into high and low expression groups by median value of mRNA expression and validated by K-M survival curves and Log-rank test. The number-at-risk cases, HRs, 95% CIs, and *P*-values were displayed accordingly. *P*-value <0.05 was considered statistically significant.

### TRANSFAC prediction of transcription factors binding

We retrieved 2000 bp upstream of transcription start sites of human GINS1, GINS2, GINS3, and GINS4 genes from UCSC Genome Browser (http://genome.ucsc.edu/cgi-bin/hgTracks). AliBaba2.1 program from the TRANSFAC public platform (http://gene-regulation.com/pub/programs/alibaba2/index.html) was used to identify potential transcription factor binding based on the above stated genomic sequence. The default setting was defined as the loose prediction condition and a stringent condition was different in Pairsim to known sites, which was set to 64 and the matrix conservation, which was set to 80%.

## Results

### Expression levels of GINS subunits are significantly up-regulated in HCC

**Figure 1:**
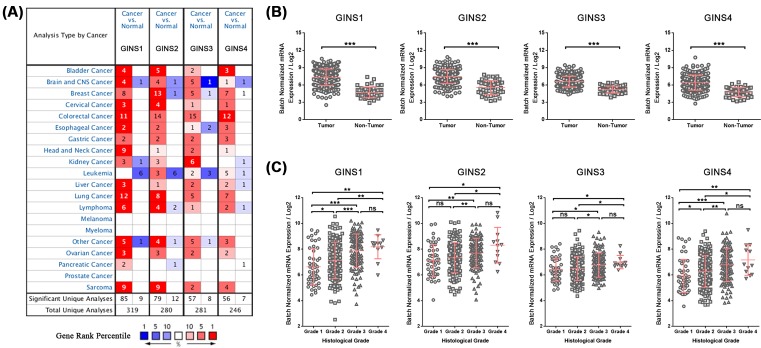
Expression levels of GINS subunits are significantly up-regulated in HCC (**A**) Expressions of GINS subunits (cancer compared with normal tissue) were analyzed with ONCOMINE database. The graphic demonstrated the numbers of datasets with statistically significant mRNA overexpression (red) or downexpression (blue) of the target genes. The number in each cell represents the number of analyses that meet the threshold within those analysis and cancer types. The gene rank was analyzed by percentile of target gene in the top of all genes measured in each research. Cell color is determined by the best gene rank percentile for the analyses within the cell. (**B**) Relative expressions of GINS subunits in HCC tumor tissues (*n*=373) and non-tumor liver tissues (*n*=50) from TCGA database. (**C**) Relative expressions of GINS subunits in HCC tumor tissues with different histological grades (Grade 1, *n*=49; Grade 2, *n*=166; Grade 3, *n*=116; Grade 4, *n*=12). *P<0.05; **P<0.01; ***P<0.001; ns, not significant.

To begin our study, we first examined the expression levels of four GINS genes in 20 types of human cancers using ONCOMINE database. All four GINS genes displayed a commonly up-regulated expression pattern in most of the cancer types analyzed except for leukemia, melanoma, myeloma, and prostate cancer (Figure 1A). Noticeably, when comparing cancer samples with normal samples in HCC, *GINS1* mRNA expression showed 5.203-fold elevation from Roessler Liver 2 dataset (*P*=6.90E-80), 4.391-fold elevation from Roessler Liver dataset (*P*=6.39E-10), and 5.203-fold elevation from Wurmbach Liver dataset (*P*=1.53E-06). Similarly, *GINS2* mRNA expression showed 1.569-fold elevation from Roessler Liver dataset (*P*=5.47E-33) in liver cancer samples. *GINS3* and *GINS4* mRNA and expressions showed 1.225- and 1.348-fold elevation from Roessler Liver 2 dataset, respectively (*P*=5.63E-25 and *P*=2.88E-26, respectively), as well as 1.185- and 1.566-fold elevation from Roessler Liver dataset, respectively, in liver cancer samples (*P*=9.05E-07 and *P*=5.68E-06, respectively) (Table 1). We also examined the expression levels of all four GINS genes in TCGA database using FireBrowse tool. Consistent with the ONCOMINE analysis, all four GINS genes were up-regulated in tumor samples of HCC from TCGA database with 6.382, 3.789, 2.442, and 2.770-fold elevation for *GINS1, GINS2, GINS3*, and *GINS4* mRNA expressions, respectively (*P*<0.001 for all) (Figure 1B). Moreover, the expression levels of all four GINS genes increased along with the severity of histological grade in HCC from TCGA database (Figure 1C). Taken together, these results suggested that all GINS subunits were commonly up-regulated in HCC, implying their potential roles in liver cancer development.

**Table 1:** Significantly up-regulated mRNA expressions of GINS subunits in HCC from Oncomine database

GINS subunits	Fold change	*P*-value	*t*test	Dataset
GINS1				
	5.203	6.90E-80	27.266	Roessler Liver 2
	4.391	6.39E-10	8.924	Roessler Liver
	5.203	1.53E-06	5.912	Wurmbach Liver
GINS2				
	1.569	5.47E-33	13.195	Roessler Liver
GINS3				
	1.225	5.63E-25	11.051	Roessler Liver 2
	1.185	9.05E-07	5.800	Roessler Liver
GINS4				
	1.348	2.88E-26	11.602	Roessler Liver 2
	1.566	5.68E-06	5.420	Roessler Liver

### Elevated expressions of GINS subunits predict poor prognosis in HCC patients, especially in male subgroup

**Figure 2:**
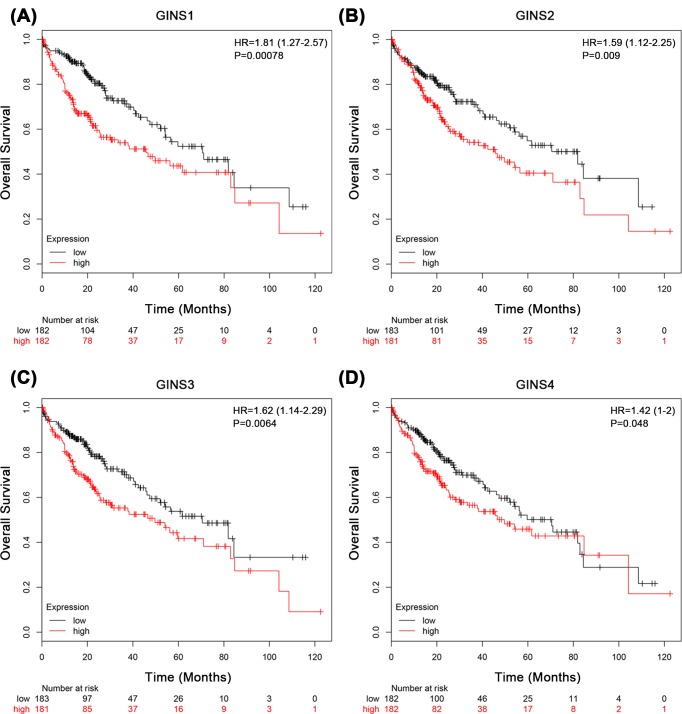
The associations between mRNA expression of each GINS subunit in tumor tissue and OS of HCC patients Each mRNA expression of GINS subunit in tumor tissue was stratified into high or low expression using the median expression value as the cut-off point. Kaplan–Meier survival curves for (**A**) GINS1 (High expression, *n*=182; Low expression, *n*=182), (**B**) GINS2 (High expression, *n*=183; Low expression, *n*=181), (**C**) GINS3 (High expression, *n*=183; Low expression, *n*=181), and (**D**) GINS4 (High expression, *n*=182; Low expression, *n*=182), and the corresponding *P*-value for Log-rank test in all HCC patients were showed.

**Figure 3:**
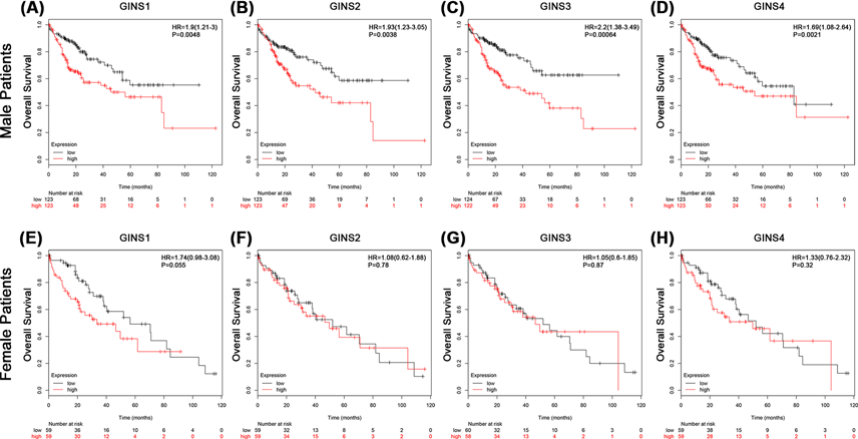
The associations between mRNA expression of each GINS subunit in tumor tissue and OS of male (upper panel) or female (lower panel) HCC patients Each mRNA expression of GINS subunit in tumor tissue was stratified into high or low expression using the median expression value as the cut-off point. Kaplan–Meier survival curves in male HCC patients for (**A**) GINS1 (High expression, *n*=123; Low expression, *n*=123), (**B**) GINS2 (High expression, *n*=123; Low expression, *n*=123), (**C**) GINS3 (High expression, *n*=124; Low expression, *n*=122), and (**D**) GINS4 (High expression, *n*=123; Low expression, *n*=123), or in female HCC patients for (**E**) GINS1 (High expression, *n*=59; Low expression, *n*=59), (**F**) GINS2 (High expression, *n*=59; Low expression, *n*=59), (**G**) GINS3 (High expression, *n*=60; Low expression, *n*=58), and (**H**) GINS4 (High expression, *n*=59; Low expression, *n*=59), and the corresponding *P*-value for Log-rank test were showed.

We next evaluated the prognostic effect of individual GINS subunit in patients with HCC using KMPlotter database. To avoid high false discovery rate, we use the median value to separate high and low groups for the survival analysis. The results showed that high mRNA expressions of GINS1 (HR = 1.81, *P*=0.00078), GINS2 (HR = 1.59, *P*=0.009), GINS3 (HR = 1.62, *P*=0.0064), and GINS4 (HR = 1.42, *P*=0.048) were significantly associated with poor overall survival (OS) in all HCC patients (Figure 2A–D). We also correlated the mRNA expressions of individual GINS subunit with survival of patient subgroups sorted by gender or histological grade in HCC. In male HCC patients, high mRNA expressions of GINS1 (HR = 1.9, *P*=0.0048), GINS2 (HR = 1.93, *P*=0.0038), GINS3 (HR = 2.2, *P*=0.00064), and GINS4 (HR = 1.69, *P*=0.021) were significantly associated with poor OS (Figure 3A–D and Table 2). However, in female HCC patients, no significant correlation was observed between mRNA expressions of GINS1 (HR = 1.74, *P*=0.055), GINS2 (HR = 1.08, *P*=0.78), GINS3 (HR = 1.05, *P*=0.87), and GINS4 (HR = 0.32, *P*=0.32) with OS (Figure 3E–H and Table 2). With respect to different histological grades, only high mRNA expression of GINS1 was significantly associated with poor OS in grade II (HR = 2.79, *P*=0.013) but not grade I (HR = 1.46, *P*=0.22) or grade III (HR = 1.75, *P*=0.063) HCC patients. No significant correlation was observed between mRNA expressions of the other 3 GINS subunits with OS of patient subgroups sorted by histological grades (Table 3). Moreover, we observed an even bigger HR value for the significant association between high mRNA expressions of GINS1 (HR = 4.67, *P*=0.0043) and GINS3 (HR = 3.41, *P*=0.019) with poor OS in grade II male HCC patients (Supplementary Table S1). Taken together, these results suggested that mRNA expression levels of GINS subunits may have prognostic prediction values in HCC patients, especially in male subgroup.

**Table 2:** Summary of the associations between of GINS subunit expression with OS by gender in HCC patients

GINS subunits	Gender	HR	95% CI	*P*-value
GINS1				
	Male	1.90	1.21–3.00	0.0048*
	Female	1.74	0.98–3.08	0.055
GINS2				
	Male	1.93	1.23–3.05	0.0038*
	Female	1.08	0.62–1.88	0.78
GINS3				
	Male	2.20	1.38–3.49	0.00064*
	Female	1.05	0.60–1.85	0.87
GINS4				
	Male	1.69	1.08–2.64	0.021*
	Female	1.33	0.76–2.32	0.32

* *P*<0.05.

**Table 3:** Summary of the associations between of GINS subunit expression with OS by histological grade in HCC patients

GINS subunits	Histological grade	HR	95% CI	*P*-value
GINS1				
	I	1.46	0.79–2.70	0.22
	II	2.79	1.20–6.49	0.013*
	III	1.75	0.96–3.18	0.063
GINS2				
	I	1.12	0.61–2.05	0.72
	II	1.75	0.79–3.90	0.16
	III	1.13	0.62–2.07	0.69
GINS3				
	I	1.61	0.87–2.98	0.12
	II	1.71	0.77–3.79	0.18
	III	1.37	0.74–2.51	0.31
GINS4				
	I	1.16	0.63–2.13	0.64
	II	2.10	0.94–4.72	0.065
	III	1.04	0.57–1.89	0.9

* *P*<0.05.

### Co-overexpression of more than two GINS subunits is correlated with shorter median survival

**Figure 4:**
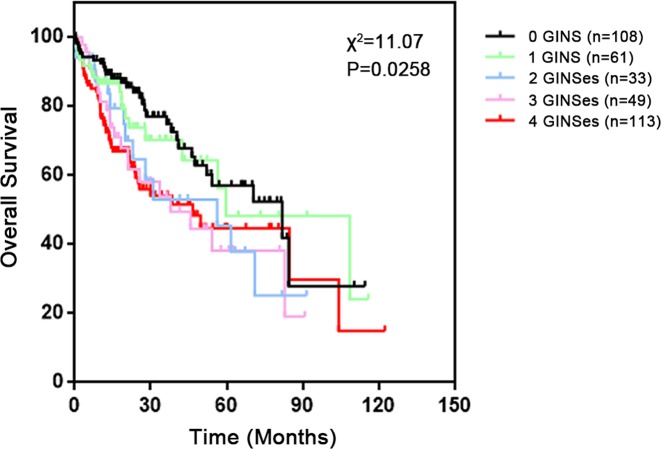
Survival analysis of HCC patients grouped by the number of overexpressing GINS subunits Kaplan–Meier survival curve for all HCC tissues stratified into five groups according to the number of overexpressing GINS subunits (0 overexpressing GINS, *n*=108; 1 overexpressing GINS, *n*=61; 2 overexpressing GINSes, *n*=33; 3 overexpressing GINSes, *n*=49; 4 overexpressing GINSes, *n*=113) and the corresponding *P*-value for Log-rank test were showed.

As overexpression of any of the GINS subunits was associated with poorer OS of HCC patients, we hypothesized that a combinatorial analysis of expression of GINS complex could provide a better prognostic prediction value for HCC. Kaplan–Meier analysis showed that HCC patients from TCGA database whose tumors overexpressed more than two GINS members had a significant shorter median survival compared with those whose tumors overexpressed two or fewer (0 GINS, 81.9 months; 1 GINS, 59.7 months; 2 GINSes, 56.2 months; 3 GINSes, 37.8 months; 4 GINSes, 46.6 months; Log-rank test, *P*=0.0258) (Figure 4). Indeed, we found that overexpression of 1 or 2 GINSes was barely significantly associated with an increased HR value, while overexpression of 3 or 4 GINSes were both statistically significantly associated with a bigger HR value (Table 4). Taken together, these results suggested that the combinatorial use of expression of three or four GINS subunits may be a reliable prognostic indicator for HCC patients.

**Table 4:** Correlation between the number of GINS subunits overexpressed and the prognosis in HCC patients

Factor	Hazard ratio	95% CI	*P*-value
GINS subunit overexpression			
0 GINS	Reference		
1 GINS	1.198	0.662–2.198	0.5420
2 GINSes	1.707	0.902–3.804	0.0939
3 GINSes	1.879	1.101–3.784	0.0238*
4 GINSes	1.896	1.218–2.987	0.0051*

* *P*<0.05.

### Expression levels of GINS subunits are highly correlated with each other

**Figure 5:**
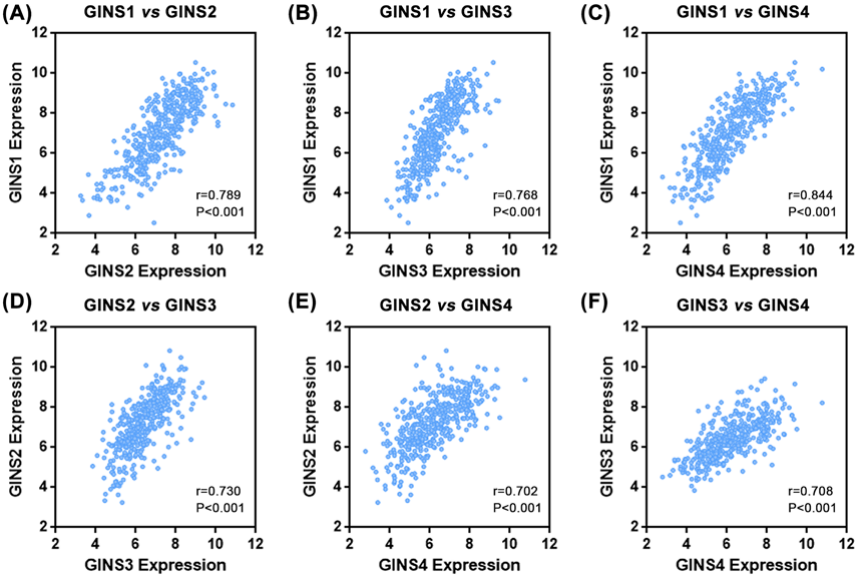
Expression levels of GINS subunits were highly correlated with each other Dot-plot graphics from analysis of cBioPortal Cancer Genomics hepatocellular carcinoma dataset (*n*=423) showed the correlation between GINS1 vs GINS2 (**A**), GINS1 vs GINS3 (**B**), GINS1 vs GINS4 (**C**), GINS2 vs GINS3 (**D**), GINS2 vs GINS4 (**E**), and GINS3 vs GINS4 (**F**). Insets indicated Spearman correlation scores and *P-*values.

As the expression of all GINS subunits were consistently up-regulated and were significantly associated with poor prognosis in HCC, we wondered whether their expressions were under the control of somewhat common regulatory element. To investigate this issue, the correlations between the expression levels of GINS subunits were examined in the TCGA dataset. As shown in Figure 5A–F, the mRNA expression levels of all the GINSes were statistically significantly associated with each other (Spearman’s correlation test, *P*<0.001). These results implied that GINS subunits may be transcriptionally regulated by some common element. Indeed, DNA sequence analysis of the 2000 bp upstream of the transcription start sites of all GINS genes using TRANSFAC tool, identified 16 potential transcription factors that may bind to those promoter sequences with a loose prediction condition (default condition) and even when searching with a stringent condition, four overlapping candidate transcription factors were found (Table 5). Specifically, these four transcription factors were C/EBPα, Oct-1, Sp1, and USF. Taken together, these results suggested that GINS subunits may be closely co-regulated in HCC by common transcription factors, but further study was needed.

**Table 5:** Summary of TRANSFAC results indicating transcription factors that may potentially bind to the promoters and regulate the expressions of all four GINS subunits under loose or stringent conditions

Loose condition	Stringent condition
AP-1	C/EBPalpha
AP-2αA	Oct-1
C/EBPα	Sp1
C/EBPβ	USF
ER	
GATA-1	
Hb	
MCM1	
NF-1	
NF-κB	
Oct-1	
RAP1	
RAR-α1	
Sp1	
TBP	
USF	

## Discussion

In the present study, we demonstrated that all GINS subunits were highly expressed in HCC. Overexpression of individual GINS subunit was all significantly associated with a poor prognosis in HCC patients, especially in male group. Expression levels of GINS subunits were highly correlated with each other as C/EBPα, Oct-1, Sp1, and USF may be the common upstream transcription factors regulating their mRNA expressions. Additionally, co-overexpression of 3 or 4 GINS subunits poses a significantly shorter median survival and higher HR value. Therefore, we propose that individual GINS subunit or GINS complex as a whole could be potential prognostic biomarkers for HCC.

When sorting the cohort by gender, the significant GINS survival association could only be observed in male patient group but not the female patient group. In prostate cancer, overexpression of Male Germ Cell-Associated Kinase (MAK), a male-specific kinase and co-activator of androgen receptor leads to increased expression level of GINS2 by mRNA profiling [23]. In addition, NCOA4, a transcriptional co-activator of androgen receptor, inhibits DNA replication origin activation by regulating CMG complex [24]. Activity of both MAK and NCOA4 are regulated in an androgen-dependent manner [25]. Thus, we hypothesize here that sexual related factors, for example androgen level, may be associated with the GINS expression in HCC patients. However, the insignificant association may merely result from a small sample number of the female patient group in our study. Therefore, more investigations are needed to address this question.

Previous studies have focussed on a prognostic role for individual GINS subunit in cancer progression [8,9,12,13,22]. Here we demonstrated that a better prognostic indicator from concurrent overexpression of 3 or 4 GINS subunits was sought in our study cohort by Kaplan–Meier survival analysis, which suggested that the combinatory use of GINS expression levels could be of clinical significance for prognosis prediction of HCC patients. Additionally, a previous study had indicated that inhibiting the expression of any one subunit of GINS complex by siRNA transfection would result in degradation of the others in HeLa cells [16]. Consistently, our study showed that expression levels of GINS subunits were highly correlated with each other or under the regulation of common transcription factors. Although the exact mechanism for co-regulation of GINS expression was yet to be defined, we provided novel findings to support the combinatory use of GINS expressions in prognostic prediction of HCC patients due to their concordance of expression and function in cancer progression.

There are limitations in our study. Our study of prognostic prediction was mainly based on the HCC patient cohort from TCGA database. Although laboratory experiment procedures could remain consistent from one database, more independent HCC patient cohorts are warranted to confirm our findings. To the best of our knowledge, this is the first report where we evaluated the prognostic values of all the GINS subunits once in HCC patients. Our study provided a whole image of the correlation between GINS expression and patient prognosis in one cancer type, which made the case more convincing that GINS activity plays an important role in the development and progression of HCC.

In conclusion, the present study has demonstrated that all GINS subunits are highly expressed in HCC and are associated with poor survival of HCC patients, especially in male patient group. The combinatory use of expression of GINS subunits could provide a better prognostic indicator for HCC patients. Expression of GINS subunits are closely correlated with each other in HCC and are predicted to be regulated by common transcription factors.

## Supporting information

**Supplementary Table 1.** Summary of the association of the association between of GINS subunit expression with OS by histological grade in male HCC patients

## Abbreviations

Cdc45: Cell Division Cycle 45
C/EBP: CCAAT/enhancer binding protein
CI: confidence interval
CMG: Cdc45–MCM helicase–GINS
GINS: go-ichi-ni-san
HCC: hepatocellular carcinoma
HR: hazard ratio
KMPlotter: Kaplan–Meier Plotter
MAK: male germ cell-associated kinase
MCM: minichromosome maintenance protein complex
NCOA4: nuclear receptor coactivator 4
OS: overall survival
TCGA: The Cancer Genome Atlas
TRANSFAC: TRANScription FACtor database
USF: Upstream Transcription Factor
