# Supplementary material for: Up-regulated and interrelated expressions of GINS subunits predict poor prognosis in hepatocellular carcinoma
Source: Biosci Rep. 2018 Dec 7;38(6):BSR20181178. doi: 10.1042/BSR20181178 (PMC6435550; doi:10.1042/BSR20181178)
Supplement: Supplementary file 1 [file bsr20181178_Supp1.pdf]

Supplementary Table 1. Summary of the associations between of GINS subunit expression with OS by histological grade in male HCC patients

GINS Subunits	Grade of Male HCC Patients	HR	95% CI	*P* Value
GINS1				
	I	1.28	0.56-2.92	0.56
	II	4.67	1.47-14.79	0.0043*
	III	1.90	0.90-4.01	0.087
GINS2				
	I	0.99	0.43-2.24	0.97
	II	2.49	0.89-6.96	0.074
	III	1.47	0.69-3.12	0.31
GINS3				
	I	1.67	0.72-3.86	0.23
	II	3.41	1.16-10.01	0.019*
	III	1.89	0.88-4.05	0.095
GINS4				
	I	0.94	0.41-2.12	0.87
	II	1.88	0.68-5.19	0.21
	III	1.52	0.72-3.20	0.27

*P<0.05
